# Improved results in proteomics by use of local and peptide-class specific false discovery rates

**DOI:** 10.1186/1471-2105-10-179

**Published:** 2009-06-12

**Authors:** Lau Sennels, Jimi-Carlo Bukowski-Wills, Juri Rappsilber

**Affiliations:** 1 Wellcome Trust Centre for Cell Biology, School of Biological Sciences, University of Edinburgh, Edinburgh, EH9 3JR, UK; 2 Centre for Systems Biology at Edinburgh, University of Edinburgh, Edinburgh, EH9 3JR, UK

## Abstract

**Background:**

Proteomic protein identification results need to be compared across laboratories and platforms, and thus a reliable method is needed to estimate false discovery rates. The target-decoy strategy is a platform-independent and thus a prime candidate for standardized reporting of data. In its current usage based on global population parameters, the method does not utilize individual peptide scores optimally.

**Results:**

Here we show that proteomic analyses largely benefit from using separate treatment of peptides matching to proteins alone or in groups based on locally estimated false discovery rates. Our implementation reduces the number of false positives and simultaneously increases the number of proteins identified. Importantly, single peptide identifications achieve defined confidence and the sequence coverage of proteins is optimized. As a result, we improve the number of proteins identified in a human serum analysis by 58% without compromising identification confidence.

**Conclusion:**

We show that proteins can reliably be identified with a single peptide and the sequence coverage for multi-peptide proteins can be increased when using an improved estimation of false discovery rates.

## Background

Current proteomic investigations have greatly expanded our ability to list proteins from complex mixtures ranging from immunoprecipitated complexes to subcellular structures [1]. The validity of the proteomic approach depends critically on a reasonable estimation of the confidence in the identified proteins. The protein inference problem [2,3] aside, proteins are identified based on the comparison of peptide fragmentation spectra to sequence databases. While a single matched peptide is sufficient to identify a protein, the identification of a second peptide for the same protein corroborates the first and greatly increases the statistical confidence. Nevertheless, proteins identified with a non-corroborated single peptide account for a considerable fraction of all proteins identified and cannot simply be disregarded.

The confidence in peptide identifications is generally estimated by interrogating the quality of match between mass spectra and peptides. False identifications are reduced through manual interrogation of peptide-spectrum matches, by applying filters created using a training data set [4], using probabilistic approaches [5-7], or relying on machine learning [8]. However, a key problem is the difficulty of determining the reliability of reported identifications as we lack a field-wide standard describing identification confidence. As a result, only experts for exactly the data interpretation method used can judge if a presented list leans towards over- or under-reporting protein identifications.

The target-decoy approach, combining the ordinary (target) database usually with an inverted (decoy) database, offers a platform-independent method to determine the confidence of protein identifications and hence addresses the standardization problem of MS-based proteomics [9-11]. The database search is performed against a concatenated database composed of target and decoy sequences. The target sequences are of such proteins that could be present in the sample while the decoy sequences are all false and normally obtained by simply inverting the target sequences. There is no sequence overlap and the probability of a random/false identification is, at least in principle, equal in both. It is not possible *a priori *to tell which target matches are false identifications. However, the frequency of false positive peptide spectrum matches is revealed by the number of decoy matches. Currently, a cut-off score is defined and adjusted until the ratio between the global count of decoy and target matches above the cut-off reaches a desired value, which is taken as the estimation of the false discovery rate (FDR) (see Choi and Nesvizhskii [12] for a detailed description).

**Figure 1:**
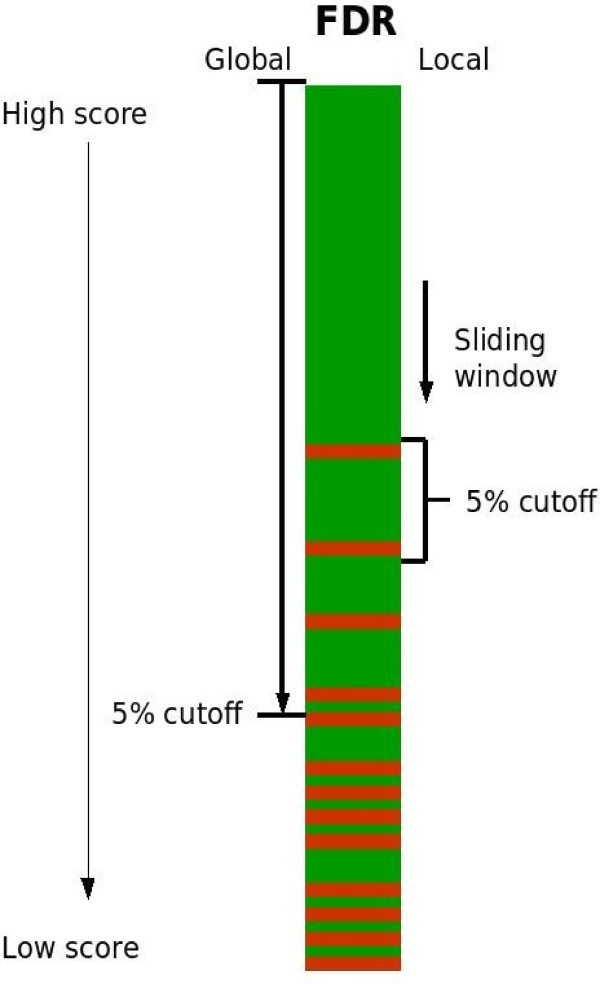
**Illustrating the principal of calculating global- and local-FDR**. The global-FDR is calculated on a list of results that rank above a given score, whilst the local-FDR is calculated on a list of results within a window surrounding a given score. In both cases the given score can be changed to obtain an FDR profile for the entire results list, or to find a particular FDR cut-off. True positives (green), false positives (red).

The target-decoy approach provides a universal expression of the identification confidence reached by a given data analysis and hence a possible path to standardization of proteomic results. The target-decoy approach generates peptide and protein lists that are very comparable using different search algorithms, as was shown recently for OMSSA, X!Tandem, Mascot, and Sequest [13]. We here complement the target-decoy approach by investigating the validity of the false-positive estimation. Furthermore we introduce an alteration to the target-decoy approach to maximize the number of correctly identified proteins while minimizing the number of false positives, even when single-peptide hits are included. To achieve this, we calculate the FDR locally within a score window (as illustrated in Figure 1) and separately consider matches to proteins alone or in groups. The local FDR calculation was previously discussed by Käll *et al*. [14] and is related to the posterior probability (probability = 1 – local FDR) as used by PeptideProphet (discussed by Choi and Nesvizhskii [15]).

## Results and Discussion

### A true-false target database serves as a control for the success of FDR estimations

We used a test system to control the selectivity of the target-decoy method. We searched the mass spectrometric data obtained by analyzing *E. coli *lysate against a non-redundant database that contained Arabidopsis and Chicken in addition to *E. coli *sequences using Mascot (for OMSSA see additional file 1). Normally, if the aim was to identify *E. coli *proteins, one would use an *E. coli*-only database as that would reduce the number of random matches, i.e. false identifications, and maximize the number of correctly identified peptides. We were, however, interested in testing how well false and correct identified peptides are discriminated from each other by the target-decoy method and thus needed a way in which to recognize the false peptide identifications. While the target-decoy approach reveals the fraction of false identifications and thus allows reducing false positives to a defined rate it does not reveal which peptides are actually false. Adding the 19-times larger Arabidopsis/Chicken sequence databases to *E. coli *enables us to obtain most of that information in our experiment. Theoretically, all false matches are random, and hence 95% (19/20) should occur to the non-E. coli part of the database making them recognizable. Importantly, we do not choose our cut-off to minimize the number of non-*E. coli *matches but merely use these false target matches to reveal the success of different ways of estimating the FDR. Note that we include only the highest scoring spectrum match for a given peptide sequence rather than including all peptide spectrum matches. This simplifies the computations and discussion presented below. We also tested taking all PSMs, as is normally done, and could not see a significant difference in the outcome of our analysis.

Aiming for a 5% FDR for matches we found a score (Mascot score minus the homology value if present else minus the identity value) threshold of 3.2, to yield the desired number of 374 decoy matches (4.8%) out of a total of 8179 matched peptides above the threshold. At the next lower cut-off (score = 1.9) the number of decoy matches exceeds our tolerated error rate (6.7%). From the equal probability of matching randomly decoy and target peptides follows that there should be 374 incorrect peptides distributed amongst the target peptide matches in relation to the relative proportion of Arabidopsis+Chicken:*E. coli *database size 19:1 (355 peptides:19 peptides). We observed 337 matches (4.3%) to non-*E. coli *peptides demonstrating that the false positive calculation is performing essentially as expected.

**Figure 2:**
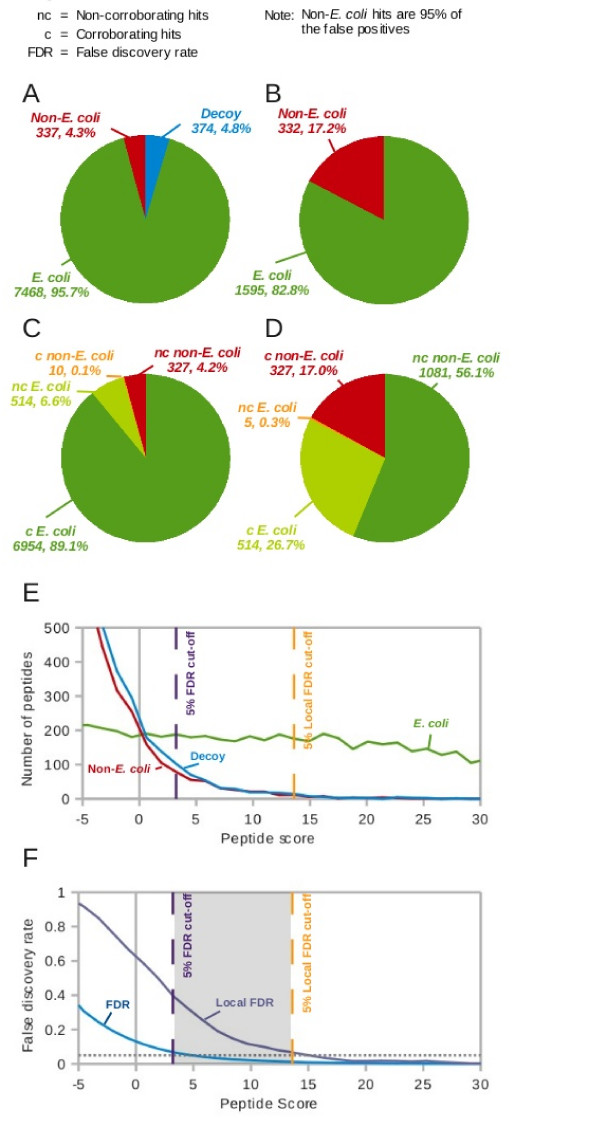
**LC-MS/MS dataset of *E. coli *lysate searched against a combined *E. coli*/non-*E. coli *target-decoy database**. Number of peptides (A) and proteins (B) identified with 5% FDR. Number of peptides (C) and proteins (D) falling into the class of corroborating (c) and non-corroborating (nc) peptide hits. (E) Distribution of *E. coli *and non-*E. coli *peptides over peptide score. FDR: number of decoy peptides at or above a score divided by the number of target peptides at or above the same score; local FDR: number of decoy peptides in a score window divided by the number of target peptides in that window. (F) Plot of the FDR and the local FDR over the peptide score. The grey zone marks the score region of those peptides that would be included as correct results according to the FDR but excluded by the local FDR. **Score is defined as Mascot score minus the homology value if present or minus the identity value**.

These 337 non-*E. coli *target peptides gave rise to 332 (17%) of our identified proteins being non-*E. coli*. The vast majority (98%) of these non-*E. coli *and thus very likely false proteins were identified with a single peptide only, while many *E. coli *proteins were identified with multiple peptides (Figure 2A, B, C, D). This summarizes into the observation that correct peptides accumulate together in proteins while false peptides tend to remain single (also known as non-random grouping [16]). Discarding all proteins with a single peptide and hence reducing the number of falsely identified proteins comes at the price of also losing a significant number of proteins that are likely correct. We hence set out to extend the target-decoy approach to more accurately reflect the FDR of the identified peptides and to increase confidence into proteins identified by a single peptide.

We next asked if the non-*E. coli *peptides scattered randomly over the list of identified peptides or clustered and could thus be removed selectively.

### Locally estimated FDR to predict probabilities for individual hits

As discussed by Käll *et al*. [14], globally estimated FDR is essentially an estimate of confidence (q-value) and is a measure of the quality of a list. However, in order to estimate the probability of individual peptide spectrum matches being false, the posterior error probability is used. We calculate this as the locally estimated FDR, in which only PSMs falling within a score window contribute to the FDR for PSMs within that window. Sliding the window over the entire list allows locally calculate FDR value to be assigned to each PSM. Since the decoy hits cluster towards lower scores, setting a cut-off for local FDR gives a much more conservative score threshold than for global FDR. This can be observed in Figures 2E and 2F, which show the decoy and target distributions and corresponding local and global FDRs over the mascot score. It is important to note that whilst it is more appropriate to consider the global FDR when working with lists of matches, we are next considering relationships between individual matches, and so use of the local FDR seems more valid.

**Figure 3:**
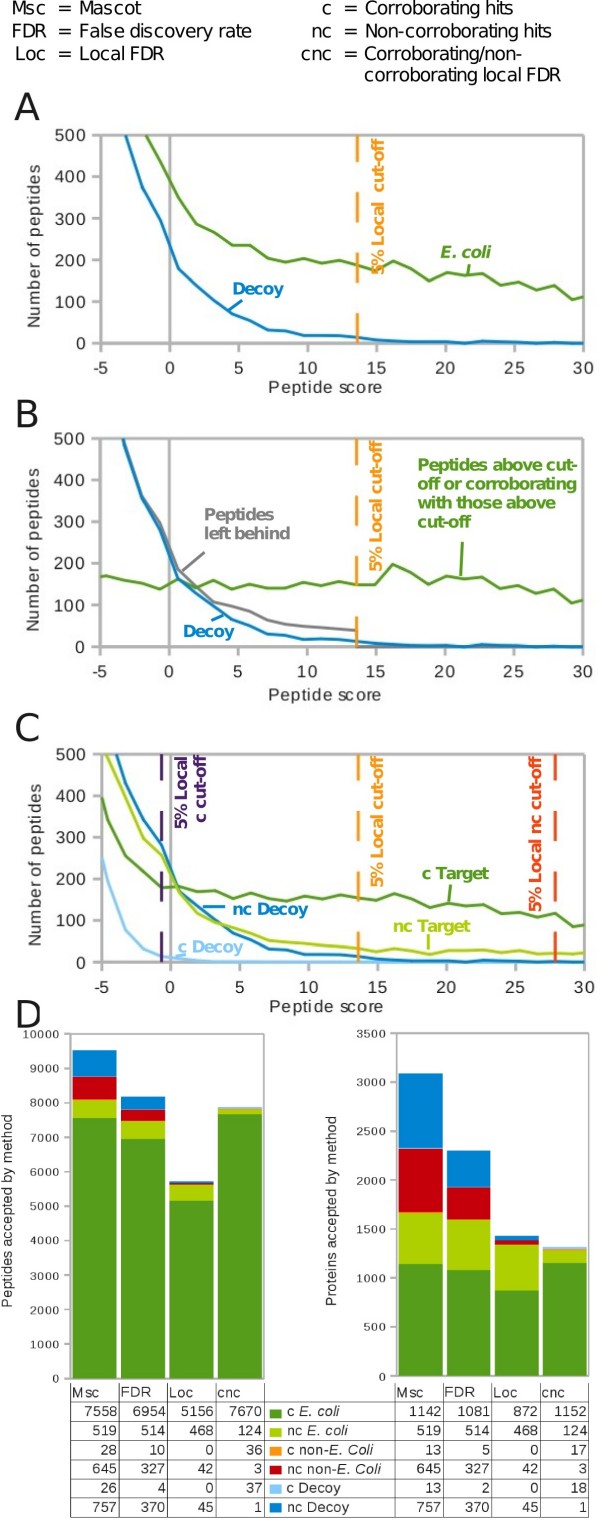
**LC-MS/MS dataset of *E. coli *lysate searched against a combined *E. coli*/non-*E. coli *target-decoy (ACE) database**. (A) Distribution of target peptides (green) and decoy peptides (blue) over the peptide score. (B) as in panel (A) but the target peptides below the score cut-off at 5% FDR split into two distributions: those matching to proteins identified by peptides above the score cut-off (green) and those that do not (left behind: grey). (C) Number of peptides (corroborated and non-corroborated) identified as a function of the score cut-off. (D) Number of peptides and proteins identified by Mascot alone (Msc), and with 5% FDR as a function of the way in which the FDR was determined: number of decoy peptides at or above the score cut-off divided by the target peptides at or above the score cut-off (FDR); number of decoy peptides in a score window divided by the number of target peptides in that window (local FDR; Loc); as local FDR but considering separately peptides that corroborate each other in matching the same protein in or above the current score window and those that do not corroborate (CNC local FDR; cnc).

Applying a cut-off to our *E. coli *dataset according a 5% local FDR, i.e. moving the calculation window from the high scoring matches down the list until the local FDR reaches 5%, resulted in 42 (0.5%) Non-*E. coli *peptides among the identified peptides. This gave 42 (2.2%) falsely identified, non-*E. coli *proteins. Using local FDR excluded many non-*E. coli *(i.e. false) peptides. However, just one increment below the cut-off, the peptides have almost the same FDR as above the cut-off. Many peptides below the cut-off are correct but nevertheless excluded as exemplified by the excess of target over decoy peptides below the cut-off (Figure 3A). Only at much lower score values did the number of target and decoy peptides equalize indicating complete random matching. The peptides below the score cut-off could potentially be critical in expanding the depth of the analysis towards low abundant proteins. The question is how to access these peptides without compromising confidence in the identified proteins.

### Peptides from below the local FDR cut-off do not result in additional correct protein identifications; they increase the sequence coverage of the already identified proteins

We divided the matches with score values below the cut-off into two classes. The first class of peptides would match to proteins already identified by peptides from above the cut-off and thus increase the sequence coverage of these proteins. The second class of peptides would add novel proteins to the list if the cut-off was lowered. Importantly, the chance of matching randomly to one of the proteins identified already is significantly smaller than the chance of matching randomly to one of the many remaining entries from the database. This is also exemplified by the behaviour of non-*E. coli *matches: 92,978 (96%) of 96,812 class two peptides are non-*E. coli *and thus false positives while it is only 432 (3.7%) of 11,654 class one peptides. Hence, class-one peptides are much more likely correct. We observe that class one peptides largely accounted for the excess of target matches found below the cut-off, while the distribution of class two peptides closely coincided with the distribution of decoy matches (Figure 3B). Lowering the cut-off by one score unit would result in the inclusion of 172 class one peptides, all of which are from *E. coli*, and 25 class two peptides – and thus 25 proteins – of which 16 (64%) are not from *E. coli*. Clearly, including additional peptides irrespective of their class by lowering the cut-off would add a large proportion of false proteins to the list. Nevertheless, correct peptides are found below the local FDR cut-off. They mostly match to proteins identified already, improving sequence coverage. However, some of these peptides will be false and we require unbiased criteria for determining what fraction of matches to upgrade onto the list of identified peptides and which to leave out.

All of the 42 non-*E. coli *proteins were identified with a single peptide and hence lack the validation by a second peptide. In effect, 8% (42) of the 510 proteins we identified with a single peptide were non-*E. coli *while this is the case for none of the 872 proteins identified with two or more peptides. This means that this FDR-based method has two deficiencies: first by excluding likely correct peptides matching to proteins identified already (corroborating) and second by including likely incorrect peptides matching proteins alone (non-corroborating). This opens the question if those two classes of matches should be treated separately for the FDR computation.

### Separating corroborating and non-corroborating matches in target-decoy analyses improves selectivity for correct identifications

The likelihood of falsely identifying a single non-corroborating matches is much higher than falsely identifying a peptide that is corroborated by matching together with other matches to the same protein. Correspondingly, identified decoy matches match to a protein much more frequently alone than together (Figure 3C). Treating both classes of matches together distorts the FDR for both. On one hand, the score cut-off is too high for corroborating matches. A consequence is the excess of target matches over decoy matches below the local FDR cut-off, described above. On the other hand, the score cut off is too low for non-corroborating matches. False, non-corroborating matches are included above the local FDR cut-off, exemplified by the large number of non-*E. coli *proteins with a single match being identified in our search. Hence, the two classes of matches should be considered individually for FDR estimation to improve selectivity for correct identifications. On some existing platforms such as Protein Prophet (Number of Sibling peptides) [7] and Spectrum Mill the obvious step is already done of allowing one to set higher score thresholds for peptides contributing to one-hit proteins than for peptides contributing to multi-hit proteins. This concept should also be implemented in the platform-spanning target-decoy approach. By applying cut-offs corresponding to 5% local FDR separately to the two match classes the vast majority of non-*E. coli *target matches were rejected in our *E. coli *analysis. Of the 127 proteins identified by a single peptide 3(2%) were non-*E. coli*. In addition, the number of corroborating *E. coli *peptides increased from 5156 to 7670 and thus the sequence coverage of the proteins was optimized without lowering confidence criteria. In fact, considering corroborating (C) and non-corroborating (NC) matches separately for local FDR (i.e. CNC local FDR) estimation and cut-off yields increased corroborating peptide and protein identifications over both FDR and local FDR methods. The proportion of *non-E. coli *to total targets among proteins identified with a single peptide is 8% but by considering these non-corroborating hits separately this percentage falls to 2%. We now can report with defined confidence the non-corroborated peptides giving rise to single-match proteins.

In order to investigate if our findings are specific for search results obtained by Mascot [5] we have conducted an equivalent analysis of the search results obtained by OMSSA [17] (see Additional File 1). We find that all trends are identical and our preference for the CNC local FDR method holds across platforms. The CNC local FDR method can thus be used to express confidence in peptide identifications in cross-platform manner. Note that our results do not support the recently reported clear superiority of OMSSA over Mascot [13] but find both programs performing comparably (1382 proteins identified with Mascot compared to 1029 proteins identified with OMSSA) with an overlap of 47% in peptides and 68% in proteins.

The concept of corroborating and non-corroborating matches can be expanded further beyond highest scoring PSM for a given peptide matching alone or in groups to proteins. This can be done by further dividing the groups or by using other features than number of matches. Such features can be charge states, modifications, sequence overlap (missed cleavage), contiguity or vicinity in the protein sequence, or even proteins falling into related functional or localization classes, e.g. being members of a complex/pathway or being membrane associated. These and other features remain to be explored.

### Comparison of the Target-Decoy approach with the confidence assignment offered by Mascot

Mascot offers a confidence assignment for its peptide-spectrum matches on a per-match basis. The algorithm includes calculation of the homology value that aims to represent the 95% limit of confidence for each match. In our analysis we use the Mascot score minus the homology value, so that if the resulting score is greater than zero, Mascot has considered this hit statistically significant.

We find the Mascot cut-off (score 0) to be in approximate agreement with the 5% global FDR cut-off (score 3.2), in the data we present here. However, 40% of identified peptides at this score value are false as is indicated by the local FDR (Figure 2F) and basically all non-corroborating peptides are falsely identified as is indicated by the numbers of non-corroborating decoy and target peptides being equal at this score (Figure 3C). It should be noted that the difference of values between global FDR and Mascot was not the same under all circumstances. For the serum data discussed below the discrepancy was negative (score -2.1, Table 1) and we have observed discrepancies of a few score units (-5 to +5) between the 5% global FDR cut-off and the Mascot cut-off using other data sets and databases.

**Table 1:** Score thresholds given by different methods of analysis.

Measurement	Score threshold in *E. coli *dataset	Score threshold in serum dataset
*Mascot*	*0*	*0*
*FDR*	*3.22*	*-2.1*
*Local FDR*	*13.6*	*7.2*
*CNC Local FDR*	*-0.67, 27.88*	*-3.6, 19.6*

Whilst there is reasonable agreement between Mascot and the target-decoy based global FDR estimation method, we can improve upon both by using the CNC local FDR. We find CNC local FDR to propose a cut-off lower than Mascot/global-FDR for corroborating-peptides and higher for non-corroborating-peptides (see Figure 3C), meaning that we accept significantly more corroborating peptides than Mascot (and global FDR) and fewer non-corroborating hits, as shown in Figure 3D. The use of CNC local FDR can thus improve even on the probabilistic data evaluation done by Mascot. Value is added on the level of peptide identification by treating single and corroborating-peptides separately. Value is also added on protein level by preventing the inclusion of a number of low confidence single-peptide hits, as basically any single-peptide protein near the cut-off used by Mascot is falsely identified, and by improving on sequence coverage.

We also investigated as a base for our target-decoy based FDR analysis the Mascot score or the Mascot E-value (data not shown). The results were practically indistinguishable to the ones obtained on the basis of the difference between Mascot score and homology value.

### Impact of FDR method on a serum analysis is significant

Human serum is a high-complexity, wide dynamic-range biological mixture of great clinical relevance. However, many serum proteins are of low abundance, making their detection challenging using current technology. Proteins present at abundance levels close to the detection limit are often detected with single, low-scoring matches. This gives the match score cut-off determined by FDR estimations a decisive impact on the final protein list. We processed a large human serum dataset[18] using FDR and corroborating/non-corroborating (CNC) local FDR estimation, to see the impact of the two methods on a dataset of this nature.

**Figure 4:**
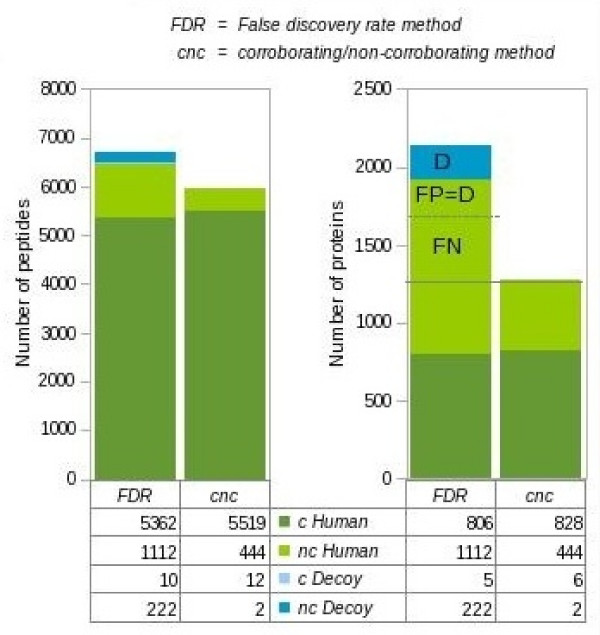
**Number of proteins identified with 5% FDR in an analysis of human serum as a function of the way in which the FDR was determined (key as in Figure 2D)**. 646 proteins were identified in addition by the current FDR approach taking proteins with one and more peptides together when compared to our CNC local FDR approach (FN+FP). Under these conditions also 222 decoy proteins are identified (D) indicating that more than 1 out of 3 additional proteins (FP) are identified on false grounds and thus would devaluate the final protein list. Our method identifies 22 additional proteins with two or more peptides. Correct proteins (FN) are missed as a result of a stringent FDR estimation and would profit from an independent protein list created from peptide identifications using a higher local FDR.

Adopting CNC local FDR increases the number of confidently identified serum proteins in our analysis by 466 (58%) from 806 to 1272 by allowing the inclusion of proteins identified with a single peptide without significantly increasing the expected number of false identifications (Figure 4). These proteins were also seen when using global FDR but mixed with a large number of false positives. Using the latter method, one therefore has to choose between accepting single peptide hits and with this a large number of false positives and rejecting single peptide hits and loosing a large number of correctly identified proteins. Using CNC local FDRs, many of the correct proteins can be identified with high confidence even though they were observed with a single peptide.

If one used the number of decoys (222) to estimate the number of false positives in the single-peptide proteins identified by the global FDR method, and subtract this number from the total (1112-222 = 890), there are about 450 additional single-peptide proteins identified by the global FDR method than by our CNC local FDR method using a 5% cut-off. Some of these 450 peptides may be additional false positives in the global FDR method that the decoy approach has failed to model, whilst the remainder will be false negatives in the CNC local FDR method, that simply did not score highly enough to meet the 5% cut-off criterion. These peptides may nevertheless be reported, albeit with a defined higher local FDR using the CNC local FDR method. Researchers can then pick biologically meaningful candidates for subsequent study or intersect the list with the results of alternative, orthogonal high through-put studies and thus exploit the information gained by mass spectrometry-based proteomics to completion.

## Conclusion

Current target-decoy methods average the probability of false identification over all reported peptides and thereby significantly increase the frequency of false identifications. We show that this leads to the effective loss of proteins identified with a single peptide. The use of a 19-times larger false-target database allows to asses the quality of the target-decoy based FDR method used, separating the cyclic redundancy of using decoy matches to calculate FDRs as well as evaluating the method of calculating FDRs. We obtain a better separation between correct and incorrect peptide spectrum matches thus minimizing false identifications, by using local FDRs to treat peptides separately that fall alone or in groups into proteins. As a result, we gain confidence into single peptide proteins and optimize the sequence coverage for multi-peptide proteins. We thus maximize the results that can be obtained with the available data at a defined FDR using the cross-platform compatible target-decoy approach. We furthermore show that the CNC local FDR method improves in confidence and number on the results obtained by Mascot and OMSSA parameters alone and that standardization does hence not compromise the quality of obtained results. This is valuable contribution towards the standardized presentation of proteomics data.

## Methods

### *E. coli *lysate

100 μg *E. coli *total soluble cell lysate was electrophoresed through Novex NuPAGE 1 mm 4–12% tris-glycine gels (Invitrogen) in MOPS buffer (Invitrogen), fixed in 50% methanol/5% acetic acid, and stained with the Colloidal Blue kit (Invitrogen). Bands were excised and processed following standard trypsin digestion procedure [19]: reduction in 100 mM DTT for 30 minutes at room temperature, alkylation with 55 mM iodoacetamide for 30 minutes at room temperature in the dark and digestion with 12.5 ng/μl trypsin (Proteomics Grade, Sigma) over night at 37°C. The supernatant was loaded onto StageTips [20,21], and peptides were eluted in 20 μl 80% acetonitrile, 0.1% trifluoroacetic. The acetonitrile was allowed to evaporate (Concentrator 5301, Eppendorf AG, Hamburg Germany), the volume of each eluate was adjusted to 5 μl with 1% trifluoroacetic acid and each sample was injected for LC-MS/MS analysis.

### LC-MS/MS analysis

The analytical platform was composed of an Orbitrap mass spectrometer (ThermoElectron) and an Agilent 1200 binary nano pump (Palo Alto, CA). C18 material (ReproSil-Pur C18-AQ 3 μm; Dr. Maisch GmbH, Ammerbuch-Entringen, Germany) was packed into a spray emitter (100 μm ID, 8 μm opening, 70 mm length; New Objectives, USA) using an air-pressure pump (Proxeon Biosystems, Odense, Denmark) to prepare an analytical column with a self-assembled particle frit [22]. Mobile phase A consisted of water, 5% acetonitrile and 0.5% acetic acid and mobile phase B of 99.5% acetonitrile and 0.5% acetic acid. The samples were loaded from a CTC PAL autosampler onto a loop and from there onto the column at 700 nL/min flow rate. The fractions were analyzed on 88 min gradients. The gradient ran with a flow rate of 300 nL/min from 0% to 20% mobile phase B in 75 minutes and then to 80% in 13 min. The mass acquisition was performed in cycles of one FT-MS (one microscan, 200 ms fill-time, 5 × 105 ion scan target, resolution 30,000, lock mass 429.0887 and 445.1200) and MS2 in the iontrap for the six most intense species (Normal scan, wideband activation, 105 ion scan target, maximum fill time 100 ms, dynamic exclusion for 180 seconds). The average length of an acquisition cycle of one MS and up to six MS2 was 1.6 sec (value obtained by averaging over 50 acquisition cycles in the middle of an analysis).

### Human serum

We took the largest of the datasets acquired on human serum proteins by Sennels at al. [18]. Sample preparation and data acquisition are described elsewhere [18]. Database-searching, data-processing, and false-positive estimation followed the procedure as is described for the *E. coli *data with the single exception being the precursor mass accuracy which uses the value published for this dataset (7 ppm) and the database being IPI human (v 3.23) in target-decoy configuration as is described for the database used for *E. coli *data.

### Database-searching and data-processing

Peaks were picked from raw-files using DTA-Supercharge (v 1.01, , peak picking parameters: initial mass tolerance 0.5, max search level 5, picking segment size 200, without internal peak picking or higher level reduction) and combined into a single peak file. The peak-data was searched against a combined Arabidopsis/Chicken/*E. coli *(ACE) sequence target-decoy database using Mascot (v 2.2, Matrix Science, London, UK) and OMSSA (v 2.1.1) [17]. Mascot was used with the parameters: mono-isotopic masses, 5 ppm on MS and 0.6 Da on MS2, ESI TRAP parameters, fully tryptic specificity, carbamidomethylation of cysteine as fixed modification, oxidation of methionine as variable modification, three missed cleavage sites allowed. For every spectrum the best matching peptide was extracted from the Mascot-output without further restriction on score or mass error to obtain the peptide-spectra match (PSM) dataset used for subsequent calculations. More than 99% of all peptide-spectrum matches, disregarding score, fell within 4 ppm of their theoretical mass showing that our 5 ppm cut-off did not significantly restrict the chance of identifying correct peptides. OMSSA was used with the parameters: mono-isotopic masses, 0.08 Da on MS and 0.6 Da on MS2, b- and y-ions considered, fully tryptic specificity, carbamidomethylation of cysteine as fixed modification, oxidation of methionine as variable modification, three missed cleavage sites allowed. The E-value was chosen to 100 to not restrict the reported results.

### Database construction

The target-decoy database was constructed from IPI *Arabidopsis thaliana *v 3.54, IPI Chicken v 3.49 and all *E. coli *sequences in the Swiss-Prot DB release 52.3 (Arabidopsis – Chicken – *E. coli *database; ACE database). For each species, **a **Markov chain model (word length = 4) was used to generate a decoy version for each sequence. The target and decoy portions of the Arabidopsis, Chicken and *E. coli *databases were combined to give the ACE database. It should be noted that the number of hits returned by Mascot for Arabidopsis and Chicken was proportional to the volume of sequence data underlining that hits to sequences of these two species are equally random. Assuming that this relationship also holds for *E. coli *and since the ratio of sequence data volume Arabidopsis + Chicken: *E. coli *is approximately 19:1, we estimate that 95% of the false positives in any search are Arabisopsis and Chicken hits. Contaminant peptides were identified by sequence identity to peptides found in the human database (human IPI v 3.55) and removed prior to FDR calculations.

### False-positive estimation

Peptides at or above a given score were counted using in-house software written in Perl . For both datasets, only peptides with more than 6 residues and a precursor mass error of 4 ppm/7 ppm (*E. coli *and *H. sapiens *serum respectively), were considered. Only non-redundant peptide-spectrum matches (PSMs) were considered. For this, the highest scoring instance of a sequence was kept while all subsequent matches were eliminated, irrespective of charge or modification. FDRs were also calculated for unique combinations of sequence, charge and modifications, but this did not result in substantial changes to the outcome described here for unique sequence alone. We therefore refrained from complicating the approach by including these extra parameters.

We are interested in estimating how many target peptides are falsely matched. Decoy peptide hits reveal the number of target peptide hits anticipated to be false for a given cut-off. However, decoy hits themselves do not contribute to the false positive rate of the reported result, as they can be clearly distinguished from target hits. The global FDR is obtained in the current implementation of the target-decoy approach [23] by dividing two times the number of decoy peptides at or above the score cut-off by the sum of the number of targets and decoys above the cut-off, which gives an estimate of the proportion of false positives among both target and decoy populations above the cut-off. In contrast, we felt that the global FDR should be estimated as the number of decoy peptides at or above the score cut-off divided by the number of target peptides at or above the score cut-off (Table 2). This gives an estimate of the proportion of false positives among the target population above the cut-off and we have used this method here. The same reasoning was also made recently [24]. Note that we have also compared our methods with the results of the decoy-incorporating method and observe similar trends throughout our analyses.

**Table 2:** A glossary of key terms, their abbreviations and formulae.

Measurement	Formula	Description
Corroborating (c) hits		Correct assignments matching together with others to a protein
Non-corroborating (nc) hits		Correct assignments matching singly to a protein
FDR (global FDR)	estimated by	Fraction of incorrect assignments above the score threshold
Local FDR	FDR for peptides with score (S):	Fraction of incorrect assignments within a score window
CNC Local FDR		Fraction of incorrect assignments within a score window, separately for assignments matching together with others (c) or singly (nc) to a protein

The local FDR was estimated as a moving average of the number of decoy peptides found in a window centred around the score, divided by a moving average of the number of target peptides with scores in the same window (Figure 1). The window width was set to 0.05 score-units wide for Mascot, using the Mascot score minus the homology threshold if present or minus the identity threshold, and also 0.05 score units for the OMSSA score, for which minus ten times the logarithm (base ten) of the OMSSA E-value was taken. The resulting distributions were then smoothed across a range of 1 score unit. We also analysed our data using the Mascot score alone, Mascot identity score (score minus identity threshold), the Mascot E-score (-10 * log(E-value)) and the OMSSA P-score (-10 * log(P-value)), but found no significant difference in the outcome, and we therefore demonstrate the most commonly used score in method development for each algorithm (OMSSA E-value, Mascot score – homology/identity threshold).

For peptide class-specific local FDRs, the cut-off for peptides matching together to a protein (corroborating hits) was determined first and then for peptides remaining alone (non-corroborating hits). First we determined whether a peptide is corroborating at a score *S *by considering all peptides at the score *S *and above, and secondly we segregated these corroborating hits from the non-corroborating hits. The local FDR was then computed as described above for each class of hits in such a way that every peptide contributed only once to the local FDR estimation, either as corroborating or non-corroborating hits.

The cut-offs for all FDR calculations were taken as the centre of the score window at which the local FDR exceeded 5% and peptides at or above this score were defined as successfully identified (Table 1). Since the Mascot score we use is the difference between the Mascot score and the homology/identity threshold, a cut-off of zero automatically gives us the peptides that Mascot would have accepted at 95% confidence. The peptides accepted by OMSSA were taken as those with and E-value less than 0.01.

#### Protein inference

Every protein identified must have at least one unique, accepted peptide. To avoid peptides being assigned twice, we ranked the peptide-spectrum matches by score and then only considered the highest scoring PSM for a given peptide. This means that every peptide occurs only once in the final results, and as such, no two proteins can be identified on the basis of the same peptide being observed. The protein this peptide is assigned to is maintained from the database search, i.e. Mascot or OMSSA, respectively. This means that we have relied on Mascot (or OMSSA) for deciding on which proteins to infer based on the peptides that were accepted by the respective FDR methods.

## Competing interests

The authors declare that they have no competing interests.

## Authors' contributions

LS conducted the initial studies. JB reproduced the results, improved the analysis and conducted all analyses shown in the manuscript. JR conceived of the study and participated in its design and coordination. All authors helped to draft the manuscript and approved the final manuscript.

## Supplementary Material

Additional file 1 **Analysis of results obtained with OMSSA**. Same analysis as shown in Figure 2 and Figure 3 but the MS/MS search was performed using OMSSA. Click here for file
