# Supplementary material for: Improved results in proteomics by use of local and peptide-class specific false discovery rates
Source: BMC Bioinformatics. 2009 Jun 12;10:179. doi: 10.1186/1471-2105-10-179 (PMC2709624; doi:10.1186/1471-2105-10-179)
Supplement: Additional file 1 — Analysis of results obtained with OMSSA. Same analysis as shown in Figure 2 and Figure 3 but the MS/MS search was performed using OMSSA. [file 1471-2105-10-179-S1.doc]

Table S1: Score thresholds for OMSSA data, given by different methods of analysis

| Measurement | Score threshold in *E. coli* dataset |
| --- | --- |
| OMSSA | 20.00 |
| FDR | 10.25 |
| Local FDR | 30.40 |
| CNC Local FDR | 3.05, 41.40 |

| 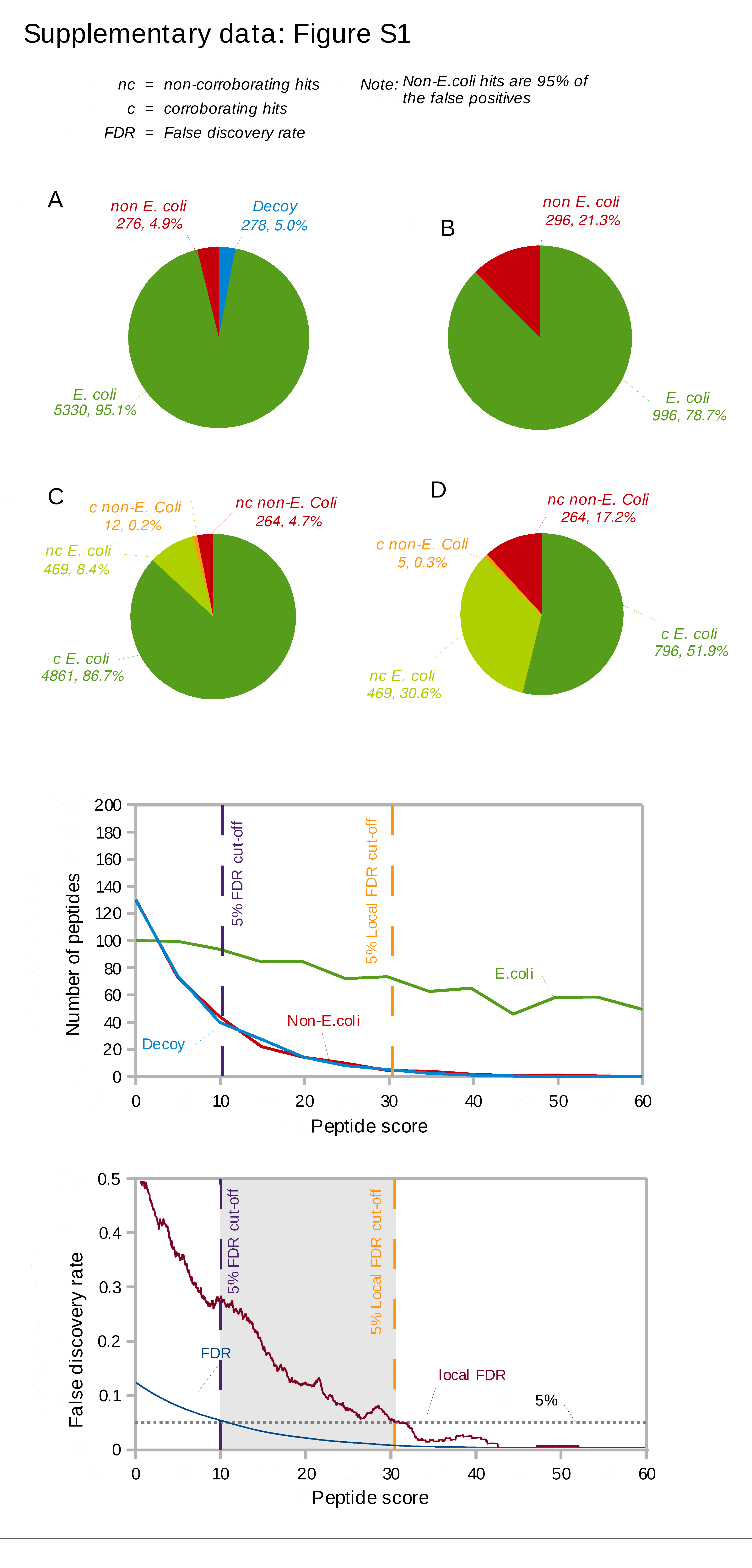 | As for Figure 2, but the MS/MS search was performed using OMSSA. LC-MS/MS dataset of *E. coli* lysate searched against a combined *E. coli*/non-*E. coli* target-decoy (ACE) database. Number of peptides **(A)** and proteins **(B)** identified with 5% FDR. Number of peptides **(C)** and proteins **(D)** falling into the class of corroborating (c) and non-corroborating (nc) peptide hits. **(E)** Distribution of *E. coli* and non*-E. coli* peptides over peptide score. FDR: number of decoy peptides at or above a score divided by the number of target peptides at or above the same score; local FDR: number of decoy peptides in a score window divided by the number of target peptides in that window. **(F)** Plot of the FDR and the local FDR over the peptide score. The grey zone marks the score region of those peptides that would be included as correct results according to the FDR but excluded by the local FDR. Score is defined as minus ten times the logarithm (base ten) of the OMSSA E-value. |
| --- | --- |

| 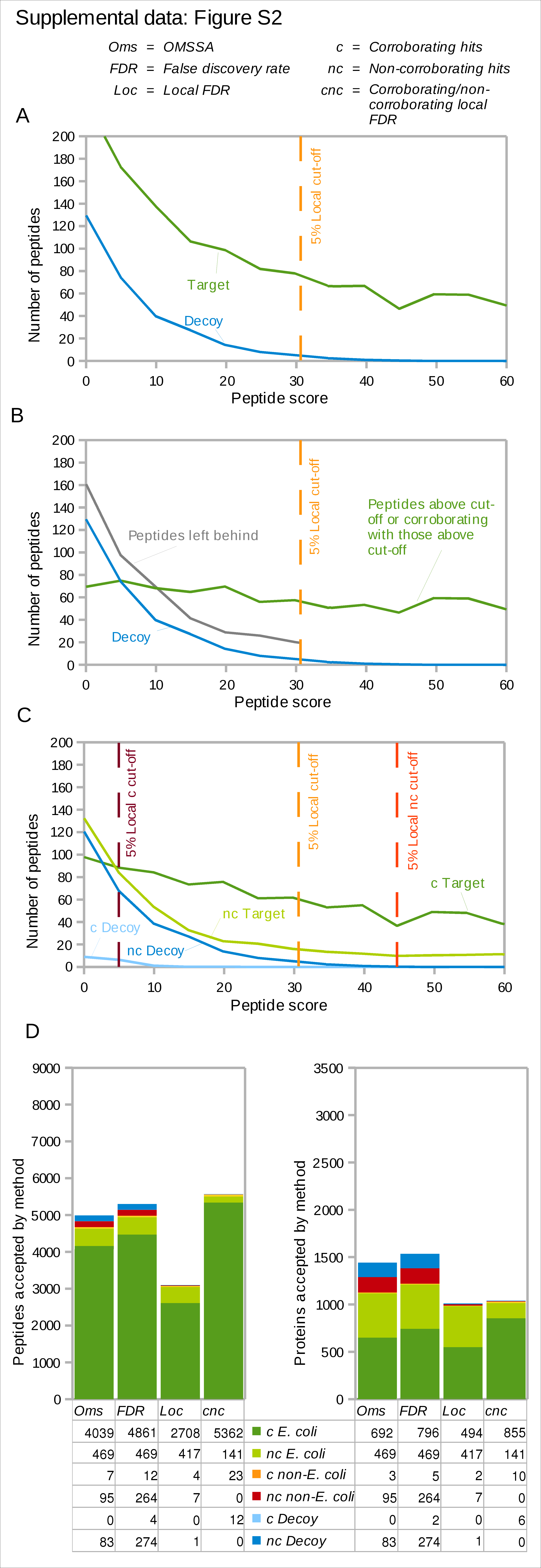 | As for Figure 3, but the MS/MS search was performed using OMSSA. LC-MS/MS dataset of *E. coli* lysate searched against a combined *E. coli*/non-*E. coli* target-decoy (ACE) database. **(A)** Distribution of target peptides (green) and decoy peptides (red) over the peptide score. **(B)** as in panel (A) but the target peptides below the score cut-off at 5% FDR split into two distributions: those matching to proteins identified by peptides above the score cut-off (green) and those that do not (left behind: grey). **(C)** Number of peptides (corroborated and non-corroborated) identified as a function of the score cut-off. **(D)** Number of peptides and proteins identified by OMSSA alone (Oms), and with 5% FDR as a function of the way in which the FDR was determined: number of decoy peptides at or above the score cut-off divided by the target peptides at or above the score cut-off (FDR); number of decoy peptides in a score window divided by the number of target peptides in that window (local FDR; Loc); as local FDR but considering separately peptides that corroborate each other in matching the same protein in or above the current score window and those that do not corroborate (CNC local FDR; cnc). |
| --- | --- |
